# Urocortin I Protects against Myocardial Ischemia/Reperfusion Injury by Sustaining Respiratory Function and Cardiolipin Content via Mitochondrial ATP-Sensitive Potassium Channel Opening

**DOI:** 10.1155/2022/7929784

**Published:** 2022-03-29

**Authors:** Wei Liu, Liping Huang, Xue Liu, Li Zhu, Yan Gu, Wei Tian, Lin Zhang, Shengli Deng, Tian Yu

**Affiliations:** ^1^Department of Anesthesiology, Affiliated Hospital of Zunyi Medical University, 563000 Zunyi, China; ^2^Department of Anesthesiology, Chengdu Fifth People's Hospital affiliated to Chengdu University of TCM, 610000 Chengdu, China; ^3^Department of Anesthesiology, Affiliated Hospital of Qingdao University, 266000 Qingdao, China; ^4^Department of Anesthesiology, Second People's Hospital of Xindu District, 610000 Chengdu, China; ^5^Department of Anesthesiology, Chongqing Health Centre for Women and Children, 404100 Chongqing, China; ^6^Department of Anesthesiology, People's Hospital of Bozhou District, 563000 Zunyi, China; ^7^Department of Anesthesiology, Zunyi Medical University, 563000 Zunyi, China

## Abstract

**Objective:**

Our experiments were aimed at probing whether urocortin I postconditioning was beneficial for maintaining the mitochondrial respiratory function and inhibiting the surging of reactive oxygen species. In addition, our experiments also intended to reveal the relationships between urocortin I postconditioning and mitochondrial ATP-sensitive potassium channel.

**Methods:**

Langendorff and MPA perfusion systems were used to establish myocardial ischemia-reperfusion injury model and cardiomyocytes hypoxia-reoxygenation injury model in rats, respectively. Isolated hearts and cardiomyocytes were randomly divided into normal group, ischemia-reperfusion/hypoxia-reoxygenation group, urocortin I postconditioning group, and 5-hydroxysolanoic acid (5-HD)+urocortin I group. At the end of balance (T_1_) and reperfusion (T_2_), cardiac functions, mitochondrial state3 respiratory, respiratory control ratio, mitochondrial respiratory enzyme activity, and mitochondrial cardiolipin content were measured. Our experiments also observed the ultrastructure of myocardium. The changes of cardiomyocyte mitochondrial permeability transition pore, mitochondrial membrane potential, reactive oxygen species, expression of apoptosis protein, and cardiomyocytes activity were detected at the end of reoxygenation.

**Results:**

The cardiac functions, mitochondrial respiratory function, and enzyme activity of the normal group were better than other three groups at T_2_, and urocortin I postconditioning group was better than the IR group and 5-HD+urocortin I group. LVEDP, +dp/dt_max_, mitochondrial respiratory function, and enzyme activity of IR group were worse than 5-HD+urocortin I group. Cardiolipin content of the normal group was higher than the other three groups at T_2_, urocortin I postconditioning group was higher than the IR group and 5-HD+urocortin I group, and 5-HD+urocortin I group was still higher than the IR group. The ultrastructure of the normal group maintained the most integrated than the other groups, IR group suffered the most serious damage, and ultrastructure of the urocortin I postconditioning group was better than the IR group and 5-HD+urocortin I group. At the end of reoxygenation, activity of mitochondrial permeability transition pore and generation of reactive oxygen species of normal group were lower than the other groups, HR group and 5-HD+urocortin I group were higher than the urocortin I postconditioning group, and 5-HD+urocortin I group was still higher than the urocortin I postconditioning group. Normal group had the highest level of mitochondrial membrane potential at the end of reoxygenation, and the urocortin I postconditioning group was higher than the HR group and 5-HD+urocortin I group. The normal group had the lowest expression level of Bax and the highest expression level of Bcl-2 at the end of reoxygenation. Urocortin I postconditioning group had lower Bax expression but higher Bcl-2 expression than the HR and 5-HD+urocortin I group. Accordingly, the normal group had the highest activity of cardiomyocytes, and the urocortin I postconditioning group was higher than the HR group and 5-HD+urocortin I group.

**Conclusions:**

Urocortin I postconditioning can protect the activity of cardiomyocytes after hypoxia-reoxygenation injury, improve the mitochondrial respiratory function, and enhance the contractility of isolated heart after myocardial ischemia-reperfusion injury. The alleviation of myocardial injury relates to the opening of mitochondrial ATP-sensitive potassium channel.

## 1. Introduction

With the rapid improvement of people's living standard, the incidence of various heart diseases is gradually increasing, which seriously threaten human health and life. Treatment of heart diseases such as coronary artery bypass grafting (CABG), percutaneous coronary intervention (PCI), and heart transplantation may inevitably induce myocardial ischemia reperfusion injury (MIRI) [1–3]. The manifestations of MIRI can be characterized as arrhythmia, cardiomyocytes apoptosis, hemodynamic disorders, and other malignant events due to the recanalization after a long period of myocardial ischemia. How to lessen MIRI is constantly a focal point in clinical research.

Urocortin (Ucn) is a mammalian peptide member of the corticotropin-releasing factor (CRF) family. Three isoforms of Ucn have been described so far, Ucn I, Ucn II, and Ucn III [4], which differ in terms of their structure, expression, and affinity to CRF receptors. Several studies have proven that the administration of Ucn before ischemia or at the onset of reperfusion can improve cardiac hemodynamic parameters, decreased the infarct size, and attenuated apoptosis [5, 6]. Ucn I is an endogenous neuropeptide which can be highly express in the heart [7]. In a study performed on the isolated rat heart, it was demonstrated that Ucn I increased left ventricular development pressure (LVDP) and maximum derivative of left ventricular [8]. Ucn I can also increase cyclic adenosine monophosphate (cAMP) and nitric oxide (NO) to relax the coronary arteries and increase coronary flow to produce anti-MIRI effects.

Mitochondria is an important organelle for energy generation. Adenosine triphosphate (ATP), which is generated by the respiratory chains located in the inner membrane of mitochondria, is the main source of energy for life activities. ATP depletion is one of the main reasons for MIRI [9]. The mitochondrial dysfunction caused by ATP depletion can further lead to the decrease of respiratory enzyme activity, increase the activity of mitochondrial permeable transition pore (mPTP) to induce the breakdown of mitochondrial membrane potential (MMP), and surge of reactive oxygen species (ROS). In addition, cardiolipin, a characteristic phospholipid of the mitochondrial inner membrane, which has close relationships with mitochondrial respiratory function and ROS [10], will suffer great damage during MIRI [11, 12]. Ucn I addition before ischemia and during reperfusion significantly restored ATP in heart tissue, which are high-energy molecules required to prevent cell apoptosis after injury.

The blockade of mPTP opening, which triggers apoptosis and cell necrosis, was clearly demonstrated [13]. mPTP has been proven to be the key structure to regulate cell survival. mPTP opening can change the permeability of mitochondria to influence mitochondrial oxidative phosphorylation process and to cause vast ROS generation. In addition, the breakdown of MMP can lead a variety of proapoptosis factors to induce cell apoptosis [14]. Therefore, how to reduce or inhibit mPTP opening during MIRI has potential protective effects. A study performed by Liu et al. has revealed that the cardioprotective effect of urocortin was associated with a decrease in reactive oxygen species (ROS) formation [15].

Latchman confirmed that urocortin I could significantly induce high expression of Kir6.2 subunit in heart mitochondrial ATP-sensitive potassium channel (mitoK_ATP_) [16]. This indicates that mitoK_ATP_ is closely related to the myocardial protective effects of urocortin I. However, whether the protective effects of urocortin I postconditioning are related to mitochondrial respiratory function and cardiolipin content are still unclear.

**Figure 1:**
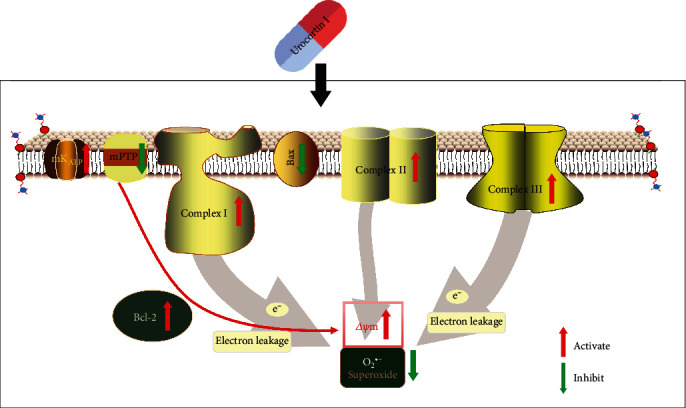
Mechanisms of urocortin I postconditioning during myocardial ischemia reperfusion injury.

In summary, our research hypothesize that Ucn I postconditioning can inhibit mPTP opening to inhibit cell apoptosis and the surge of ROS. The decrease of ROS reduces its attack on cardiolipin; thus, the normal respiratory functions of mitochondria can be sustained. Once mitochondrial respiratory functions are maintained, the ATP generation during MIRI can be guaranteed and the normal hemodynamics can be sustained. These are the possible mechanisms of urocortin I postconditioning during MIRI (Figure 1).

## 2. Materials and Methods

### 2.1. Animal and Surgical Procedures

All experiments used 16- to 20-week-old male specific pathogen-free (SPF) rats (body weight: 250 to 300 g) obtained from the Third Military Medical University experimental animal center (Chongqing, China). The rats were maintained under specific pathogen-free conditions and a 12 h light/dark cycle, with free access to food and water. All animals received humane care in accordance with the Guide for the Care and Use of Laboratory Animals published by the National Institutes of Health.

**Figure 2:**
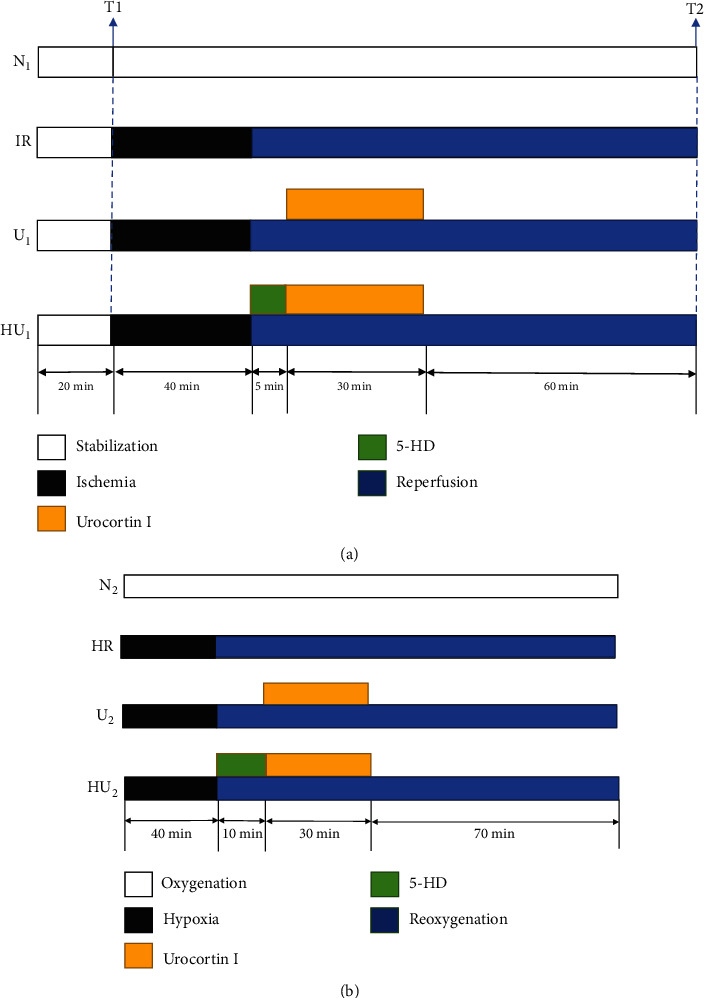
Perfusion protocol. (a) N_1_ group was perfused with 37°C K-H solution continuously for 155 min. IR group was perfused with 4°C St. Thomas cardioplegia after 20 min 37°C K-H solution perfusion and was kept ischemia at 32°C for 40 min and then reperfused with 37°C K-H solution for 95 min. U_1_ group was reperfused with 37°C K-H solution that contained urocortin I for 30 min after ischemia and then reperfused with 37°C K-H solution for 65 min. HU_1_ group was reperfused with 37°C K-H solution that contained 5-HD for 5 min before urocortin I administration, and the rest procedures were the same as U_1_ group. (b) N_2_ group was continuously cultured in the 37°C-incubator for 150 min. HR group was reoxygenated for 110 min after 40 min hypoxia. U_2_ group was reoxygenated for 10 min after 40 min hypoxia then cultured in the medium containing urocortin I for 30 min and reoxygenated for 70 min. HU_2_ group was cultured in the medium that contained 5-HD for 10 min after 40 min hypoxia, and the rest procedures were the same as the U_2_ group.

Langendorff isolated perfusion system was used to isolate rat heart. one percent amobarbital sodium (40 mg/kg) and anticoagulation with heparin (250 U/kg) were used to anesthetize rats by intraperitoneal injection. Rat was fixed on the operating table after satisfactory anesthesia. We used 75% alcohol to disinfect the rat skin; then, we cut open the abdominal wall and diaphragm along the xiphoid process to fully expose the heart. Rat heart was cut out along the root of the aorta and immediately put it into a precooled 4°C K-H solution. We used ophthalmic forceps to clamp each side of the aorta and fixed the isolated heart on the needle of Langendorff perfusion device and perfused 37°C oxygenated K-H solution. Then, we cut open the pulmonary artery and left atrial appendage and inserted the self-made rubber balloon into the left ventricle through the mitral valve orifice. After the above steps were completed, we connected Powerlab/8SP biological function experiment system and maintained the perfusion pressure at 70-80 mmHg. LVEDP was kept at 4-8 mmHg by adjusting the balloon size and position. All steps were completed in 2 min. The isolated heart for the follow-up experiments should meet three criteria after 20 min 37°C K-H solution perfusion: (1) heart rate (HR) >250beats/min, (2) left ventricular developed pressure (LVDP) >80 mmHg, and (3) ventricular premature beat <2beats/min. Those hearts which did not meet the standards were abandoned. Isolated hearts were randomly divided into normal group (N1 group), ischemia-reperfusion group (IR group), urocortin I postconditioning group (U_1_ group), and urocortin I postconditioning+5-hydroxysolanoic acid (5-HD) group (HU_1_ group). The N_1_ group was perfused with 37°C K-H solution continuously for 155 min. The IR group, U_1_ group, and HU_1_ group were all perfused with 4°C St.Thomas cardioplegia for 40 min to simulate MIRI process after completing the 20 min perfusion of 37°C K-H solution. Then, the IR group was reperfused with 37°C K-H solution for 95 min, the U_1_ group was reperfused with 37°C K-H solution that contained urocortin I for 30 min and reperfused with 37°C K-H solution for 60 min, the HU_1_ group was reperfused with 37°C K-H solution that contained 5-HD for 5 min before urocortin I administration and reperfused with 37°C K-H solution for 60 min (Figure 2(a)). The final concentrations of urocortin I and 5-HD were 10^−8^ mol/L and 10^−4^ mol/L, respectively.

MPA perfusion system was used to isolate cardiomyocytes. The system was soaked in 75% ethanol for 30 min and irrigated with sterile ultrapure water for three times in advance. Laminin was tiled and covered the bottom of the cell culture format and put the format into a 37°C-incubator containing 95%*O*_2_ + 5%*CO*_2_ for 30 min. The process of anesthesia and operation was the same as above. The rat heart was cut off and put into a 37°C-calcium solution and gently squeezed for 2-3 times to drain the remaining blood. The heart was cut out and fixed on the MPA device and was perfused with 37°C oxygenated calcium solution for 2 min, EGTA for 4 min, and enzyme digestion solution for 12-15 min successively. Then, the heart was cut open and put into a sterile flask, and 5 mL of digestion solution containing 1% BSA was added into the flask. We shook the flask in a 37°C constant temperature water bath with oxygen 5 minutes at a time. Supernatant was filtered by a 200 mu nylon filter screen and collected into a sterile centrifuge tube and placed the tube in the 37°C bath and then let the supernatant settle naturally. All procedures mentioned above were repeated 4 to 5 times. We collected the sediment and the sediments were myocytes. Sediment was washed 3 to 4 times with enzyme elution and M199 medium. We took out the cell culture format in the incubator and removed the laminin. Then, the cardiomyocytes were evenly tiled in the culture format with the density of 10^4^/cm^2^ and the M199 medium was also added. The format was put into the incubator for 4 h. Then, the cardiomyocytes were cultivated in the 37°C-incubator for 24 h after the M199 medium was replaced. Cardiomyocytes were randomly divided into normal group (N_2_ group), hypoxia/reoxygenation group (HR group), urocortin I postconditioning group (U_2_ group), and urocortin I postconditioning+5-HD group (HU_2_ group). The N_2_ group was continuously cultured in the 37°C-incubator for 150 min. The HR group was reoxygenated for 110 min after 40 min hypoxia. The U_2_ group was reoxygenated for 10 min after 40 min hypoxia and then cultured in the medium containing urocortin I for 30 min and reoxygenated for 70 min. The HU_2_ group was cultured in the medium that contained 5-HD for 10 min after 40 min hypoxia, and the rest procedures were the same as the U_2_ group (Figure 2(b)).

### 2.2. Cardiac Functions

Powerlab/8SP biological function experiment system was used to record HR, LVDP, LVEDP, and maximum dp/dt (dp/dt_max_) at the end of balance (T_1_) and reperfusion (T_2_).

### 2.3. Mitochondrial Extraction

Isolated hearts at T1 and T2 were immediately put into mitochondrial separation medium to wash out the residual K-H solution. Redundant vascular tissue was cut off, and then, the heart was put into a tube, mitochondrial separation medium was added, and then prepared the heart into suspension. Suspension was centrifugated for 10 min. We extracted the supernatant and centrifugated it at 3600*g* for 15 min. Precipitate was extracted, and the equal amount of mitochondrial separation medium was added to make myocytes resuspend; then, we centrifugated the medium at 1000*g* for 5 min. Supernatant was collected and centrifugated at 5500*g* for 10 min, and the precipitates were mitochondria. All operations were carried out on the surface of ice at 4°C.

### 2.4. Mitochondrial Protein Quantification

10 *μ*L mitochondrial sample solutions were extracted from each group. Our experiment used BSA as the standard protein. All procedures were carried out according to the Bradford protein concentration test kit produced by Beyotime Biotechnology (Shanghai, China). 10 *μ*L of standard protein was extracted after the BSA fully was dissolved; then, separation medium was added to make the final concentration to 0.5 mg/mL. 0, 1, 2, 4, 8, 12, 16, 20 *μ*L of standard protein solution were added to 96-well plates, respectively. To make the volume to 20 *μ*L, we added separation medium into each hole. We extracted 10 *μ*L of mitochondrial sample solution and diluted it with 750 *μ*L separation medium. We extracted 20 *μ*L of the diluted solution and added it into the sample hole. We added 200 *μ*L of G250 dye solution to each hole and placed the plates at room temperature for 3 min. The absorbance of the solution at 595 nm wavelength was measured by using TECAN SUNRISE multifunctional microplate reader. The protein concentration of mitochondrial samples was calculated according to the standard curve.

### 2.5. Mitochondrial Respiratory Function

We used the Lufthansa oxygen electrode (Hansatech, England) to test. The total volume of the reaction solution was 1000 *μ*L. 900 *μ*L mitochondrial respiration measurement medium was added and set the reaction temperature at 37°C; then, the electromagnetic stirrer was started and ran for 30 min. 50 *μ*L mitochondrial suspension with the concentration of 1 mg/mL was added into the oxygen electrode and recorded the curve of oxygen consumption for 20-30 s. Then, 1 *μ*L rotenone with the final concentration of 2*μ*mmol/L and 10 *μ*L succinic acid of 5 mmol/L were added after the oxygen consumption curve kept stable. The curve of oxygen consumption was recorded for 1 min when mitochondria entered State4 respiration. When the slope of the oxygen consumption curve kept stable, mitochondria entered State3 respiration; then, we added 9 *μ*L ADP with the final concentration of 100 *μ*mol/L into the reaction chamber and recorded the oxygen consumption curve. Respiratory control rate (RCR) was equal to State3/State4. Rotenone, a respiratory chain inhibitor, needed to be added when succinic acid was used as a substrate to prevent electrons from flowing back from complex II to complex I.

### 2.6. Respiratory Enzyme Activity

Lufthansa oxygen electrode (Hansatech, England) was used. Mitochondria were accepted and freeze-thawed for 3 times at 20°C and -80°C to prepare mitochondrial subunits. Total volume of the reaction solution was 1000 *μ*L, 900 *μ*L NADH-OX, and Cyt-OX, and Suc-OX measuring media were added, respectively. The reaction temperature was controlled at 37°C. Then, we added 50 *μ*L mitochondrial suspension with the concentration of 1 mg/mL and recorded the oxygen consumption curve for 5-10 min.

### 2.7. Total Phospholipid Extraction

The mitochondrial suspension was added to cardiolipin extraction at the volume ratio of 1 : 10. The mixed solution was kept standing for 20 min and then centrifugated at 1000*g* for 3 min. We extracted the precipitate and added CaCl_2_ to the concentration of 0.05 mol/L. The solution was centrifugated at 1000*g* for 10 min after 1 min standing. Then, we extracted the chloroform phase and blew it in a 40°C-water bath with nitrogen until the solvent was just evaporated. After the addition of 0.15 mL phospholipid diluent, then we stored the solution at -20°C in a closed, dark and nitrogen-containing condition. The samples were analyzed by high performance liquid chromatography (HPLC). Except for the 40°C-water bath, other steps were carried out in the 0-4°C light-avoided and air-isolated ice bath.

### 2.8. Cardiolipin

Agilent 1260 series chromatographic system and stainless-steel normal phase chromatographic column were used (Dalian, China, the column number was E2623376). Washing conditions are as follows: the detector was DAD; the detection wavelength was 206 nm and reference wavelength was 360 nm; the flow rate of mobile phase was 1 mL/min; and the column temperature was 30°C. The standard cardiolipin with the same concentration was injected several times in 6 days, and different concentrations were injected repeatedly every 4 hours in the same day to calculate the within-day and intraday precision. Qualitatively, the analysis was referred to the retention time of standard cardiolipin under the same condition and cardiolipin content was expressed as the ratio of cardiolipin content converted from the peak area integral to mitochondrial protein content.

### 2.9. Cardiolipin Standard Curve and Sample Cardiolipin Determination

Standard cardiolipin solution samples with concentration of 2000 ng/10 *μ*L, 1000 ng/10 *μ*L, 500 ng/10 *μ*L, 125 ng/10 *μ*L, and 62.5 ng/10 *μ*L were prepared with phospholipid diluent. HPLC was used to detect the peak area and draw a standard curve to analyze its correlation. Peak area of the 10 *μ*L phospholipid solution to be tested was detected by HPLC. The content of sample phospholipids was calculated according to the standard curve.

### 2.10. Myocardial Ultrastructure

1 mm^3^ size of left ventricular myocardium was taken from each group at T_2_ and fixed it in the 4°C-electron microscope solution for 4 h; then, 0.1 mol/L phosphate buffer was used to wash the solution for three times every 2 h. 1% 4°C osmium acid was used to fix it for 2 h, and then, the sample was dehydrated under different ethanol concentrations and embedded with epoxy resin. Sections were observed after staining.

### 2.11. Mitochondrial Permeable Transition Pore

All procedures were carried out according to the fluorescent detection kit for mitochondrial membrane channel pore of living cells purchased from the GENMED Pharmaceutical Technology (Shanghai, China). Fluorescence changes were observed at 488 nm excitation wavelength and 505 nm emission wavelength by inverted phase contrast microscope, and the relative fluorescence unit (RFU) was measured at the same excitation and emission wavelength by a fluorescence enzyme labeling instrument. The reduction of green fluorescence and RFU indicated the enhanced activity of mPTP.

### 2.12. Mitochondrial Membrane Potential

Operations were carried out according to the JC-1 test kit purchased from Beyotime Biotechnology (Shanghai, China). Red fluorescence was observed at 540 nm excitation wavelength and 580 nm emission wavelength by laser scanning confocal microscope, and the red or strong red fluorescence indicated the MMP was normal. The green fluorescence was observed at 488 nm excitation wavelength and 525 nm emission wavelength, and the green indicated MMP was destroyed; RFU was measured at the same excitation and emission wavelength by a fluorescence enzyme labeling instrument. The ratio of red RFU to green RFU was used to reflect the change of MMP. If MMP was damaged, the ratio reduced.

### 2.13. Reactive Oxygen Species

Operations were carried out according to the active oxygen detection kit purchased from Beyotime Biotechnology (Shanghai, China). ROS production was observed by an inverted phase contrast microscope at 488 nm excitation wavelength and 525 nm emission wavelength, and RFU was measured at the same excitation and emission wavelength by a fluorescence enzyme labeling instrument. The enhanced green fluorescence and higher RFU indicated the increase of ROS production.

### 2.14. Bax and Bcl-2 Expression

RIPA solution was added for cracking after myocytes were collected. The solution was centrifuged at -4°C, and the supernatant was extracted. BSA method was used to measure the total protein content to determine the sample amount. Bax rabbit polyclonal antibody with 1 : 6000 dilution and Bcl-2 rabbit polyclonal antibody with 1 : 2000 dilution were added for the first antibody reaction after denaturation electrophoresis, membrane transferring, and sealing of the protein. Then, the sample was incubated at room temperature for 12 h. Goat anti-rabbit IgG labeled with horseradish peroxidase with 1 : 10000 dilution was added for the second antibody reaction and incubated for 60 min. The samples were imaged by a chemiluminescence imaging system, and Image J software was used to analyze the gray value of the strip. The ratio of target protein strips to the internal parameter *β*-actin strip was used to reflect the expression level of the target protein.

### 2.15. Cardiomyocytes Viability

0.25% trypsin was added into the cell plate and gently shook it to make the adherent myocytes resuspend again. Then, the trypsin was removed and added with the culture medium into the plate carefully. 100 *μ*L solution was extracted and tiled in a translucent and black edge 96-well plate. The number of myocytes in each hole was about 1000. 10 *μ*L CCK-8 solutions was added into each hole and put it into the 37°C-incubator containing 5%*CO*_2_ + 95%*O*_2_ for 2 h. The absorbance value (OD) at the wavelength of 450 nm was detected by a fluorescence enzyme labeling instrument. The higher the OD value was, the more live the myocytes were.

### 2.16. Statistical Analysis

Statistical significance was evaluated by one-way ANOVA. If the variances were followed, the normal distribution LSD-test was used. Otherwise, Dunnett's T3 was used. Paired *t*-test was used to analyze the difference between data at T1 and T2. All data were expressed as *mean* ± *SD*. A probability value of *p* < 0.05 was considered statistically significant. All statistical analyses were performed via SPSS 17.0 software.

## 3. Results

### 3.1. Cardiomyocytes Isolated from Adult SD Rats

**Figure 3:**
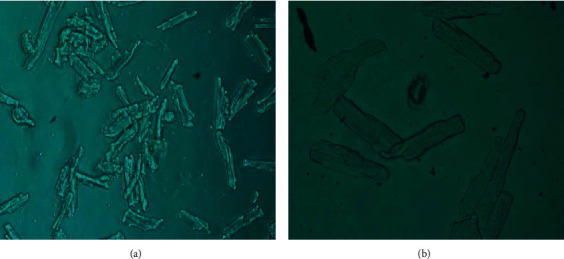
Morphology of normal isolated cardiomyocytes under microscope after 24 h culture. (a) cardiomyocytes under the magnification of 100 times. (b) cardiomyocytes under the magnification of 400 times.

As shown in Figure 3 rod-shaped or rectangle-like well-grown cardiomyocytes could be seen under the magnification of 100 times, 15%-20% of cardiomyocytes had spontaneous contraction, and the rhythm varied from 5 to 15 times/min. The rest cardiomyocytes were static, and the number of round or quasiround dead myocytes was small (Figure 3(a)). The striated muscle of cardiomyocytes whose end was ladder or rod-shaped could clearly be seen under the magnification of 400 times ( Figure 3(b)).×400.

### 3.2. Urocortin I Maintained Myocardial Ultrastructure after Ischemia Reperfusion Injury

**Figure 4:**
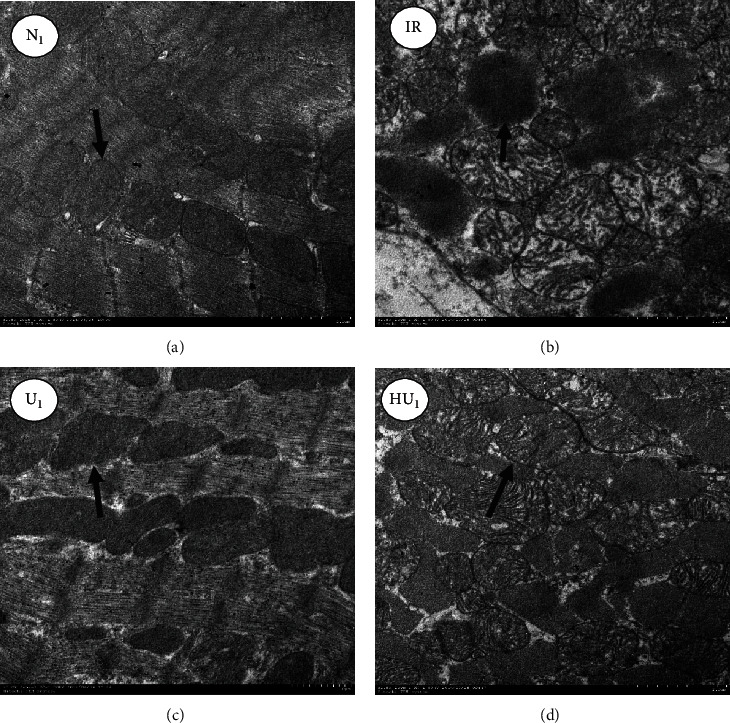
Ultrastructural of myocardium under electron microscope. (a) Myocardium in the normal group. (b) Myocardium in the IR group. (c) Myocardium in the urocortin I group. (d) Myocardium in the urocortin I+5-HD group. The ultrastructure of myocardium was observed under the magnification of 20000 times. IR: ischemia reperfusion; 5-HD: 5-Hydroxydecanoic acid sodium. The arrow indicates mitochondria.

The myocardial ultrastructure in the N1 group was normal, the arrangement of myofilaments was neat, and no obvious dissolution or rupture was found, and the sarcomere could clearly be seen. The shape of mitochondria was complete and orderly arranged. The number of mitochondria was more, and the crista membrane was closely connected (Figure 4(a)). The ultrastructure in IR group was seriously damaged, and the myofilaments were dissolved or even broken. Mitochondria were clearly swollen, and the arrangement was disordered. In addition, the number of mitochondria suffered an obvious reduction. The cristae membrane space of mitochondria was widened and broken, and the sarcoplasmic reticulum was highly expanded (Figure 4(b)). The ultrastructure in the U_1_ group was clear, and the arrangement of myofilaments remained orderly. Only a part of the myofilaments and sarcomere space were widened or dissolved, and most mitochondria were complete in shape and more in number. The cristae membrane was visible, and no dissolution or rupture was found (Figure 4(c)). The ultrastructure in the HU_1_ group was partially remained, and the myofilaments were partially dissolved or broken. The sarcomere space was widened. The mitochondria were slightly swollen, and the arrangement was disordered and the number was relatively reduced. (Figure 4(d)).

### 3.3. Cardiac Functions Were Sustained by Urocortin I

**Figure 5:**
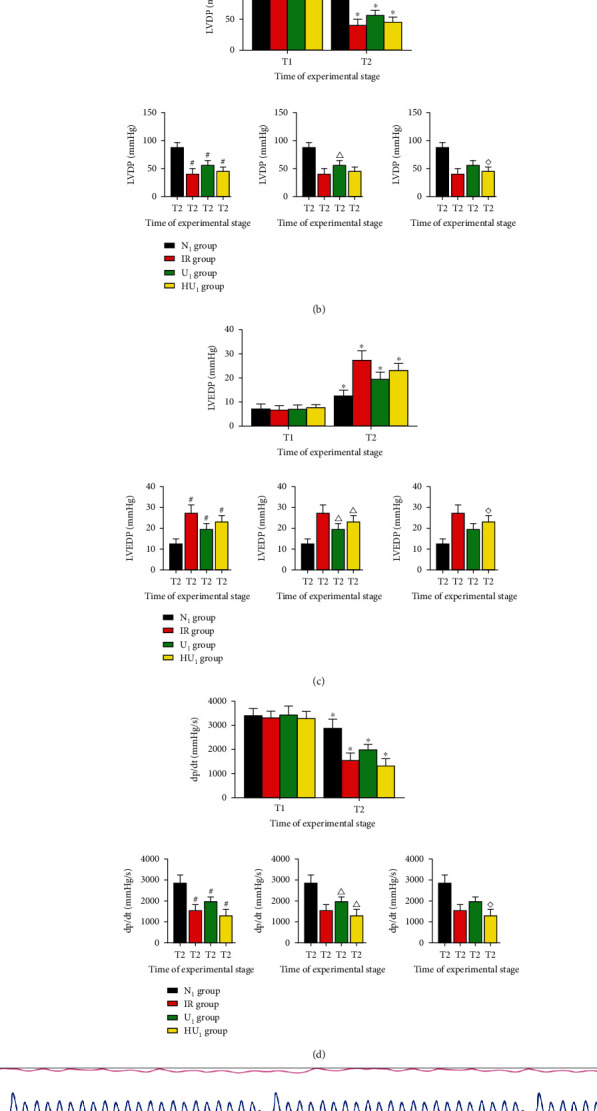
Cardiac functions after MIRI. After 40 min ischemia, rat hearts were subjected to MIRI. At the end of reperfusion, cardiac functions were evaluated by hemodynamic testing. (a) Changes of HR after MIRI (*n* = 12 in each group). ^∗^*p* < .05, ^#^*p* < .05, ^△^*p* < .05, ^◇^*p* < .05. (b) Changes of LVDP after MIRI (*n* = 12 in each group). ^∗^*p* < .05, ^#^*p* < .05, ^△^*p* < .05, ^◇^*p* < .05. (c) Changes of LVEDP after MIRI (*n* = 12 in each group). ^∗^*p* < .05, ^#^*p* < .05, ^△^*p* < .05, ^◇^*p* < .05. (d) Changes of dp/dt after MIRI (*n* = 12 in each group). ^∗^*p* < .05, ^#^*p* < .05, ^△^*p* < .05, ^◇^*p* < .05. (e) Hemodynamic data of isolated heart obtained from Langendorff device. MIRI: myocardial ischemia-reperfusion injury; HR: heart rate; LVDP: left ventricular development pressure; LVEDP: left ventricular end diastolic pressure; T_1_: at the end of balance; T_2_: at the end of reperfusion; ^∗^: intragroup comparison with T_1_; ^#^: compared with N_1_ group; ^△^: compared with the IR group; ^◇^: compared with the U_1_ group.

To determine whether urocortin I had positive effects on cardiac function, our in vitro experiments collected cardiac function. As shown in Figure 5, there were no significant differences between the groups in cardiac function at T_1_. Due to IRI, cardiac functions at T_2_ were all worse than those at T_1_. At T_2_, HR of the N_1_ group were better than the IR group (264.67 ± 12.99 vs. 215.33 ± 14.98, *p* < 0.05), U_1_ group (264.67 ± 12.99 vs. 240.83 ± 8.01, *p* < 0.05), and HU_1_ group (264.67 ± 12.99 vs. 214.50 ± 12.61, *p* < 0.05). LVDP of the N_1_ group at T_2_ were better than the IR group (87.83 ± 8.52 vs. 40.67 ± 9.46, *p* < 0.05), U_1_ group (87.83 ± 8.52 vs. 56.33 ± 8.09, *p* < 0.05), and HU_1_ group (87.83 ± 8.52 vs. 45.50 ± 7.74, *p* < 0.05). LVEDP and +dp/dt_max_ of the N_1_ group at T_2_ were also better than the IR group (12.50 ± 2.43 vs. 27.33 ± 3.88, 2863.35 ± 384.36 vs. 1304.29 ± 304.88, *p* < 0.05), U_1_ group (12.50 ± 2.43 vs. 19.50 ± 2.74, 2863.35 ± 384.36 vs. 1980.14 ± 216.09, *p* < 0.05), and HU_1_ group (12.50 ± 2.43 vs. 23.17 ± 2.79, 2863.35 ± 384.36 vs. 1544.95 ± 294.62, *p* < 0.05). HR, LVDP, LVEDP, and +dp/dt_max_ of U_1_ group at T_2_ were better than the IR group and HU_1_ group. LVEDP (27.33 ± 3.88 vs. 23.17 ± 2.79) and +dp/dt_max_ (1304.29 ± 304.88 vs. 1544.95 ± 294.62, *p* < 0.05) of the IR group were worse than the HU_1_ group at T_2_, but no significant difference was found in HR and LVDP between the IR group and HU_1_ group.

### 3.4. Mitochondrial Respiratory Function and Enzymes' Activity Were Maintained by Urocortin I

**Figure 6:**
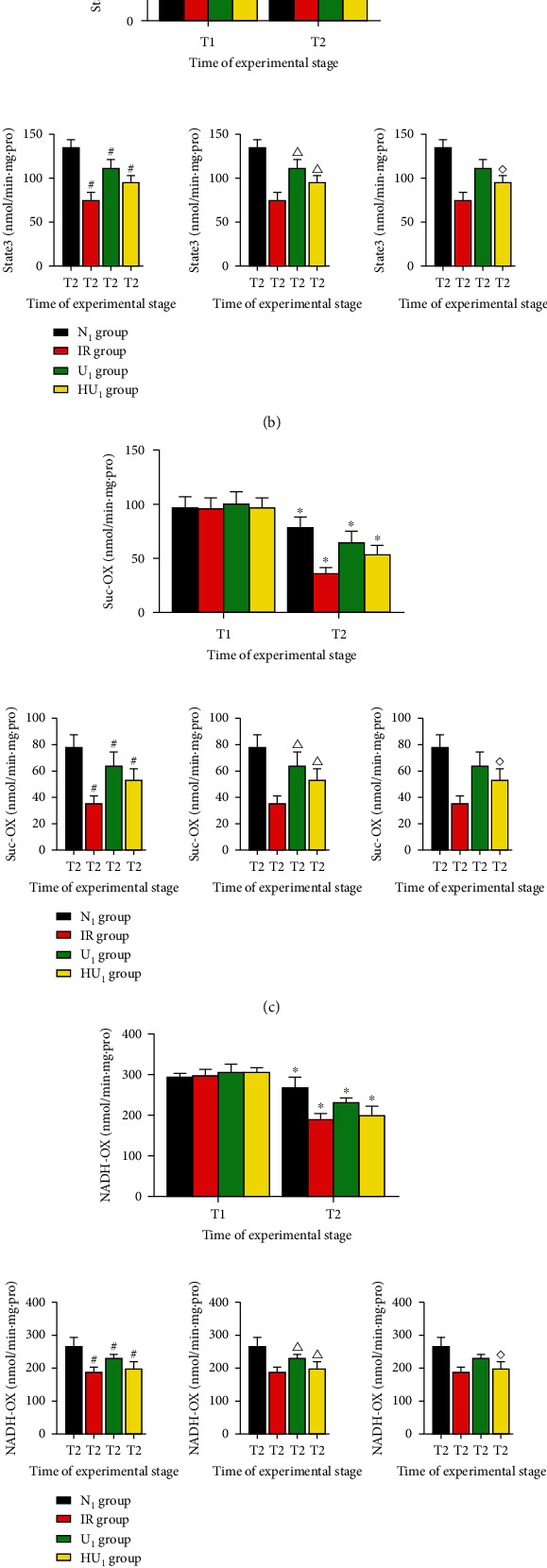
Mitochondrial respiratory function and respiratory enzymes activity after MIRI. After 40 min ischemia, rat hearts were subjected to MIRI. At the end of reperfusion, mitochondrial respiratory function and enzymes were determined by Lufthansa oxygen electrode. (a) Changes of RCR after MIRI (*n* = 12 in each group). ^∗^*p* < .05, ^#^*p* < .05, ^△^*p* < .05, ^◇^*p* < .05. (b) Changes of State3 after MIRI (*n* = 12 in each group). ^∗^*p* < .05, ^#^*p* < .05, ^△^*p* < .05, ^◇^*p* < .05. (c) changes of Suc-OX after MIRI (*n* = 12 in each group). ^∗^*p* < .05, ^#^*p* < .05, ^△^*p* < .05, ^◇^*p* < .05. (d) changes of NADH-OX after MIRI (*n* = 12 in each group). ^∗^*p* < .05, ^#^*p* < .05, ^△^*p* < .05, ^◇^*p* < .05. (e) Changes of Cytc-OX after MIRI (*n* = 12 in each group). ^∗^*p* < .05, ^#^*p* < .05, ^△^*p* < .05, ^◇^*p* < .05. MIRI: myocardial ischemia-reperfusion injury; RCR: respiratory control rate; NADH-OX: nicotinamide adenine dinucleotide phosphate oxidase; SUC-OX: succinate oxidase; Cytc-OX: cytochrome-c oxidase; T_1_: at the end of balance, T_2_: at the end of reperfusion; ^∗^: intragroup comparison with T_1_; ^#^: compared with the N_1_ group; ^△^: compared with the IR group; ^◇^: compared with the U_1_ group.

Our in vitro experiments found that urocortin I had favorable effects on respiratory function, activity of mitochondrial respiratory enzymes, and cardiolipin content. As shown in Figure 6, there was no significant difference in State4 in each group at T_1_ and T_2_. We also found no significant difference in State3 and RCR of each group at T_1_. Due to IRI, activity of respiratory enzyme of each group at T_1_ was better than those at T_2_ (*p* < 0.05 or *p* < 0.01). At T_2_, State3 of the N_1_ group was better than the IR group (134.33 ± 8.72 vs. 74.25 ± 9.11, *p* < 0.05), U_1_ group (134.33 ± 8.72 vs. 110.55 ± 9.87, *p* < 0.05), and HU_1_ group (134.33 ± 8.72 vs. 94.71 ± 7.48, *p* < 0.05). In addition, RCR of the N_1_ group at T_2_ was better than the IR group (1.90 ± 0.11 vs. 1.09 ± 0.09, *p* < 0.05), U_1_ group (1.90 ± 0.11 vs. 1.62 ± 0.08, *p* < 0.05), and HU_1_ group (1.90 ± 0.11 vs. 1.48 ± 0.08, *p* < 0.05). As to enzyme activity, Suc-OX of the N_1_ group at T_2_ was better than the IR group (78.67 ± 9.65 vs. 35.87 ± 5.68, *p* < 0.05), U_1_ group (78.67 ± 9.65 vs. 64.55 ± 10.48, *p* < 0.05), and HU_1_ group (78.67 ± 9.65 vs. 53.65 ± 8.44, *p* < 0.05). Similarly, NADH-OX of the N_1_ group was better than the IR group (268.67 ± 26.45 vs. 189.98 ± 13.88, *p* < 0.05), U_1_ group (268.67 ± 26.45 vs. 232.74 ± 9.15, *p* < 0.05), and HU_1_ group (268.67 ± 26.45 vs. 200.65 ± 21.39, *p* < 0.05). CtyC-OX of the N_1_ group was better than the IR group (85.48 ± 4.45 vs. 43.66 ± 4.59, *p* < 0.01), U_1_ group (85.48 ± 4.45 vs. 73.99 ± 4.92, *p* < 0.01), and HU_1_ group (85.48 ± 4.45 vs. 52.84 ± 3.99, *p* < 0.01). Compared with the IR group, State3, RCR, and enzyme activity of the U_1_ group and HU_1_ group was better. State3, RCR, and enzyme activity of the U_1_ group was better than the HU_1_ group. Interestingly, our experiment still found significant difference in State3, RCR, and enzyme activity between the IR group and HU_1_ group.

### 3.5. Urocortin I Inhibited the Decrease of Cardiolipin Content

**Figure 7:**
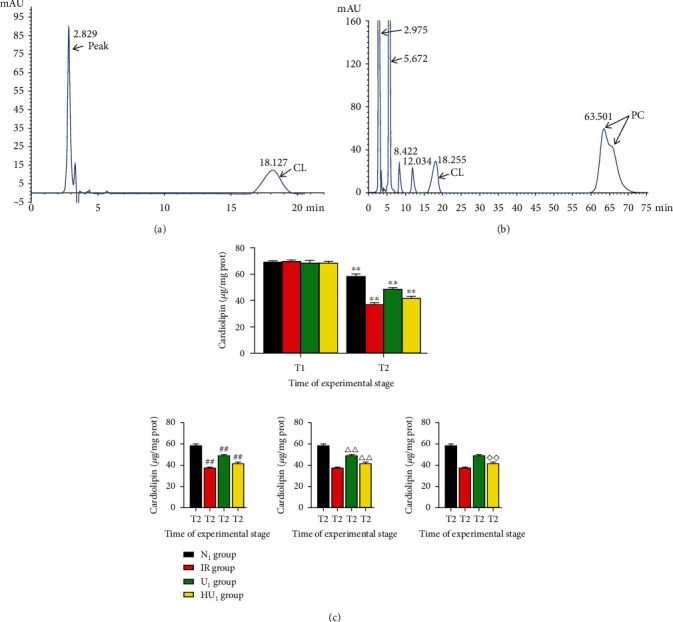
Cardiolipin content after MIRI. (a) Standard chromatogram of cardiolipin: cardiolipin concentration was 500 ng/10 *μ*L, and the injection volume was 10 *μ*L . (b), Chromatogram of myocardial mitochondrial phospholipid sample to be tested: the incoming sample was myocardial mitochondrial phospholipid extraction liquid, and the injection volume was 10 *μ*L.After 40 min ischemia, rat hearts were subjected to MIRI. At the end of reperfusion, cardiolipin content was determined by HPLC. (c) and (a) Cardiolipin content at T_1_ and T_2_. (*n* = 12 in each group). ^∗∗^*p* < .01. (b) Cardiolipin content at T_2_ in different group. (*n* = 12 in each group). ^##^*p* < .01, ^△△^*p* < .01, ^◇◇^*p* < .01. MIRI: myocardial ischemia-reperfusion injury; HLPC: high performance liquid chromatography; 5-HD: 5-Hydroxydecanoic acid sodium; T_1_: at the end of balance; T_2_: at the end of reperfusion; ^∗^: intragroup comparison with T_1_; ^#^: compared with N_1_ the group; ^△^: compared with the IR group; ^◇^: compared with the U_1_ group.

As shown in Figure 7, the within-day and intraday precision was 1.01% and 3.32%, respectively. The results met the standard of the experiment. The linear regression equation was *y* = 0.351*x* + 19.468, and the correlation coefficient was 0.9998. The concentration of standard cardiolipin was 500 ng/10 *μ*L, and the injection volume was 10 *μ*L (chromatogram of the standard sample is shown in Figure 7(a)). The volume of the sample cardiolipin to be tested was 10*μ*L (chromatogram of the sample to be tested is shown in Figure 7(b)). Due to IRI, cardiolipin content of all groups at T_2_ was lower than T_1_. At T_2_, cardiolipin content of the N_1_ group was higher than the IR group (58.31 ± 1.51 vs. 37.30 ± 0.89, *p* < 0.01), U_1_ group (58.31 ± 1.51 vs. 48.89 ± 0.97, *p* < 0.01), and HU_1_ group (58.31 ± 1.51 vs. 41.51 ± 1.49, *p* < 0.01). Compared with IR group, cardiolipin content of the U_1_ group and HU_1_ group was higher (*p* < 0.01). Cardiolipin content of the U_1_ group was higher than the HU_1_ group. In addition, cardiolipin content of the HU_1_ group was still higher than the IR group (*p* < 0.01) (Figure 7(c)).

### 3.6. Activity of mPTP and Surging of ROS Were Inhibited by Urocortin I, and the Stability of MMP Was Maintained by Urocortin I

**Figure 8:**
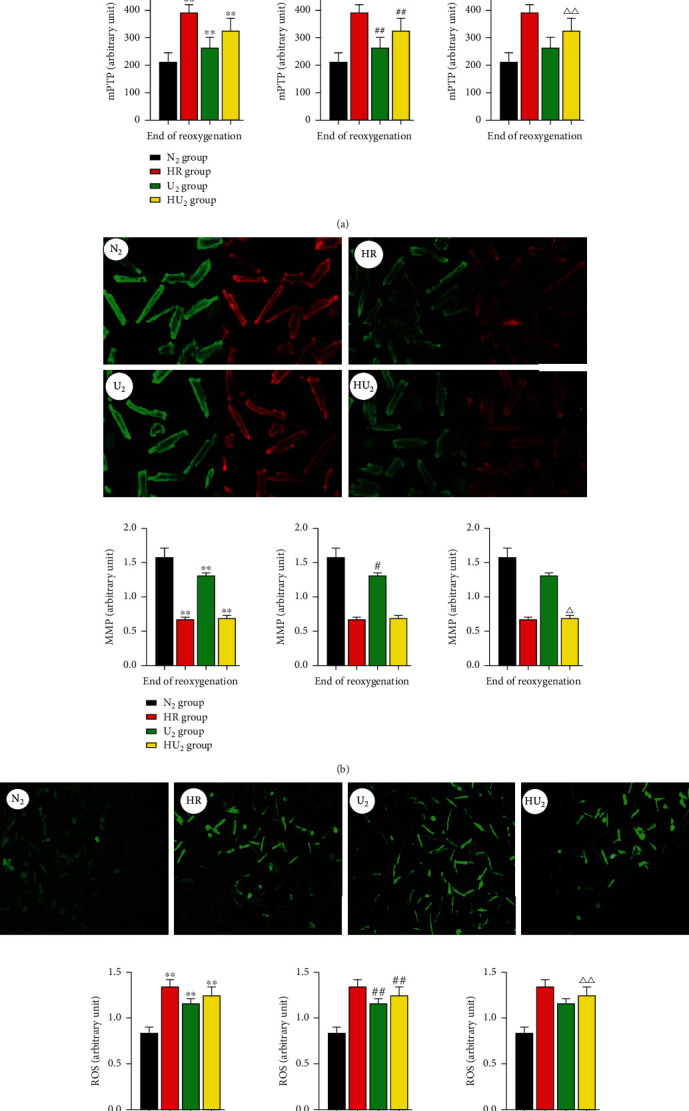
Relative changes of indexes on cellular level after HRI. After 40 min anoxia, cardiomyocytes were subjected to HRI. At the end of reoxygenation, change of mPTP, MMP, and ROS generation was determined by fluorimetry. (a) Relative change of mPTP activity (*n* = 12 in each group). ^∗∗^*p* < .01, ^##^*p* < .01, ^△△^*p* < .01. (b) Relative decrease of MMP (*n* = 12 in each group). ^∗∗^*p* < .01, ^#^*p* < .05, ^△^*p* < .05. (c) Relative generation of ROS (*n* = 12 in each group). ^∗∗^*p* < .01, ^##^*p* < .01, ^△△^*p* < .01. HRI: hypoxia reoxygenation injury; mPTP: mitochondrial permeable transition pore; MMP: mitochondrial membrane potential; ROS: reactive oxygen species; ^∗^: compared with the N_2_ group; ^#^: compared with the HR group; ^△^: compared with the U_2_ group.

As shown in Figure 8, at the end of reoxygenation, activity of mPTP of the N2 group was lower than the HR group (211.24 ± 35.21 vs. 390.82 ± 28.93, *p* < 0.01), U_2_ group (211.24 ± 35.21 vs. 262.93 ± 38.37, *p* < 0.01), and HU_2_ group (211.24 ± 35.21 vs. 326.53 ± 44.99, *p* < 0.01) (Figure 8(a)). Similarly, ROS fluorescence intensity of the N2 group was lower than the HR group (0.84 ± 0.06 vs. 1.34 ± 0.08, *p* < 0.01), U_2_ group (0.84 ± 0.06 vs. 1.15 ± 0.05, *p* < 0.01), and HU_2_ group (0.84 ± 0.06 vs. 1.25 ± 0.09, *p* < 0.01) (Figure 8(c)). MMP fluorescence intensity of the N2 group was higher than the HR group (2.31 ± 0.36 vs. 0.28 ± 0.05, *p* < 0.01), U_2_ group (2.31 ± 0.36 vs. 1.04 ± 0.06, *p* < 0.05), and HU_2_ group (2.31 ± 0.36 vs. 0.29 ± 0.06, *p* < 0.05) (Figure 8(b)). Compared with the HR group, activity of mPTP and ROS fluorescence intensity of the U2 group and HU2 group was lower, but MMP fluorescence intensity was higher. The U_2_ group had lower activity of mPTP and ROS fluorescence intensity but higher MMP fluorescence intensity than the HU_2_ group. We still found significant difference in activity of mPTP and ROS fluorescence intensity between the HR group and HU_2_ group. However, there was no significant difference in MMP fluorescence intensity between the HR group and HU_2_ group.

### 3.7. Urocortin I Inhibited the Protein Expression Level of Bax and Promoted the Protein Expression of Bcl-2 and Maintained Cardiomyocyte Viability

**Figure 9:**
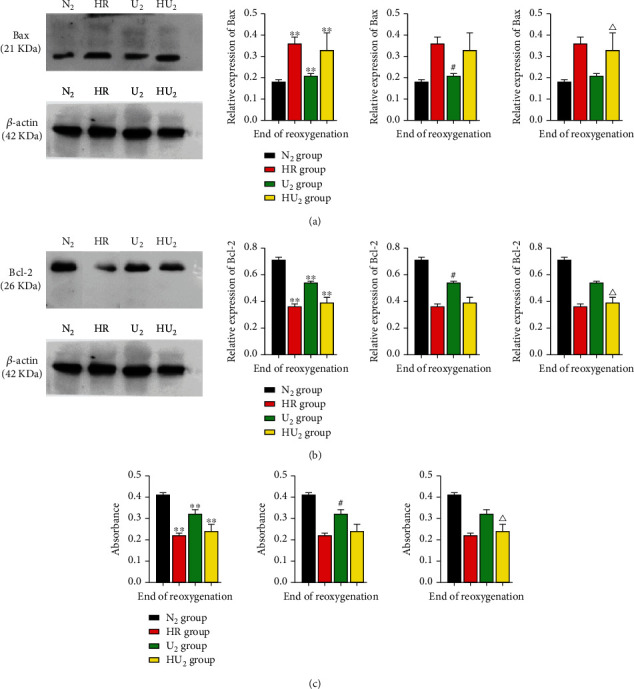
Relative expression of apoptosis proteins and cardiomyocyte viability. After 40 min anoxia, cardiomyocytes were subjected to HRI. At the end of reoxygenation, expression of apoptosis proteins was determined by Western blot, and cardiomyocyte viability was determined by fluorimetry. (a) Relative expression of Bax (*n* = 12 in each group). ^∗∗^*p* < .01, ^#^*p* < .05, ^△^*p* < .05. (b) Relative expression of Bcl-2 (*n* = 12 in each group). ^∗∗^*p* < .01, ^#^*p* < .05, ^△^*p* < .05. (c) Change of cardiomyocyte viability (*n* = 12 in each group). ^∗∗^*p* < .01, ^#^*p* < .05, ^△^*p* < .05. HRI: hypoxia reoxygenation injury; ^∗^: compared with the N_2_ group; ^#^: compared with the HR group; ^△^: compared with the U_2_ group.

To determine the effects of urocortin I postconditioning on cardiomyocyte apoptosis, our study detected the expression of Bax, Bcl-2, and cell viability. As shown in Figure 9, at the end of reoxygenation, Bax expression of the N2 group was lower than the HR group (0.18 ± 0.01 vs. 0.36 ± 0.03, *p* < 0.01), U_2_ group (0.18 ± 0.01 vs. 0.21 ± 0.01, *p* < 0.05), and HU_2_ group (0.18 ± 0.01 vs. 0.33 ± 0.08, *p* < 0.05) (Figure 9(a)). However, Bcl-2 expression of the N2 group was higher than the HR group (0.71 ± 0.02 vs. 0.36 ± 0.02, *p* < 0.01), U_2_ group (0.71 ± 0.02 vs. 0.54 ± 0.01, *p* < 0.05), and HU_2_ group (0.71 ± 0.02 vs. 0.39 ± 0.04, *p* < 0.05) (Figure 9(b)). Compared with HR group, Bax expression of the U_2_ group and HU_2_ group was lower, but Bcl-2 expression was higher. The U2 group had lower Bax expression but higher Bcl-2 expression than the HU_2_ group. We found no significant difference of Bax and Bcl-2 expression between the HR group and HU_2_ group. The cardiomyocytes viability was consistent with the results of apoptosis protein. The viability of cardiomyocytes of the N_2_ group was better than the HR group (0.41 ± 0.01 vs. 0.22 ± 0.01, *p* < 0.01), U_2_ group (0.41 ± 0.01 vs. 0.32 ± 0.02, *p* < 0.05), and HU_2_ group (0.41 ± 0.01 vs. 0.24 ± 0.03, *p* < 0.05). The viability of cardiomyocytes of HR group was worse than the U_2_ group and HU_2_ group. The viability of cardiomyocytes of the U_2_ group was better than the HU_2_ group. We found no significant difference between the HR group and HU_2_ group in the viability of cardiomyocytes (Figure 9(c)).

## 4. Discussion

Nowadays, Ischemic cardiomyopathy has become a serious threat to the safety of human life. Our experiments provide evidence that urocortin I postconditioning can produce cardiac protective effects through the maintaining of mitochondria respiratory function and activity of respiratory enzymes after MIRI. In addition, urocortin I postconditioning can regulate the expression of cardiomyocyte apoptosis protein and enhance contractility of isolated heart. Our study demonstrates that the cardiac protective effects of urocortin I postconditioning are related to the opening of mitoK_ATP_ channel. Therefore, urocortin I may have great potential protection against MIRI, and exogenous urocortin I maybe an important strategy for patients who have ischemic heart diseases.

Sufficient supply of ATP is the basis of human activities. 80%-90% of ATP is produced through mitochondrial respiratory chains and ATP synthetase. The main respiratory chains include NADH and succinic acid oxidative respiratory chain. NADH and FADH2 are dehydrogenated through respiratory chains. The energy released in the process of electron transmission can transport H^+^ to the inner mitochondrial space and stores the energy in the form of ATP through oxidative phosphorylation [17, 18]. State3 and State4 are the most important respiratory types of mitochondria. State3 reflects the stimulating efficiency of ADP on oxidative respiratory, and State4 mainly reflects the oxidative function of respiratory chain without ADP. RCR, which is referred to the ratio of State3 and State4, is an important index to evaluate the respiratory function and structural integrity of mitochondria. MIRI may lead to energy imbalance, which is bound to the dysfunction of mitochondrial respiratory function [19]. The respiratory dysfunction caused by MIRI led to the increased consumption of high-energy phosphate bonds, and the reduction of RCR and ATP generation during MIRI would further aggravate mitochondrial respiratory dysfunction [20]. In addition, complexes I and III in high reduction state will consume more energy and cause the surge of ROS [21]. As a key factor of MIRI, ROS can oxidize unsaturated fatty acids such as cardiolipin to generate more ROS and the dysfunction of mitochondrial oxidative phosphorylation induced by MIRI let a mass of electrons escape and promote the surge of ROS [22].

Our experiments found that urocortin I postconditioning could maintain mitochondrial RCR and respiratory enzyme activity such as NADH-OX, Cyt-OX, and Suc-OX to maintain the integrity of mitochondrial and inhibit oxidative phosphorylation dysfunction during MIRI, thus sustain the normal respiratory function of mitochondria to generate sufficient ATP to improve contractility of isolated heart. In addition, urocortin I postconditioning could reduce ROS generation to maintain normal content of cardiolipin to improve mitochondrial respiratory function. When we added 5-HD, a mitoK_ATP_ channel blocker, myocardial protective effects of urocortin I were weakened in varying degrees. Several researches have proven that 5-HD alone had no protective activity of pre- and postconditioning [23, 24].

MitoK_ATP_ channel, which locates in the inner membrane of mitochondria, was first found in guinea pig cardiomyocytes by Japanese scholar Noma. It is a selective voltage-independent potassium channel regulated by ATP and ADP. This channel is normally closed, but it can be activated under the conditions of ischemia or energy depletion [25]. mPTP plays an important role in stabilizing MMP and maintaining oxidative phosphorylation to produce ATP. mPTP opening during MIRI enables protons to pass freely through the inner membrane of mitochondria and causes the breakdown of MMP, which will further induce the disorder of oxidative phosphorylation and the reduction of ATP. In addition, mPTP opening can increase the release of proapoptosis factors to induce apoptosis [26]. It has been proved that apoptosis is also an important pathological process and a main form of cell death during MIRI [27]. Bcl-2 family is one of the key regulators in the apoptosis signal transduction pathway. The ratio of Bcl-2 to Bax determines the survival of the cells. The higher the ratio is, the less apoptosis will be, otherwise, the apoptosis will be heavier [28].

Our results showed that urocortin I postconditioning could inhibit mPTP opening and MMP breakdown through the opening of mitoK_ATP_ channel. It has been proved that the opening of mPTP was a prerequisite for the change of MMP [29]. Therefore, urocortin I could stabilize MMP by inhibiting the opening of mPTP. Besides, our experiments found that the ratio of Bcl-2 to Bax of the HR group was worse than that of the U_2_ group, and the viability of cardiomyocytes of the U_2_ group was better than the HR group. The results demonstrated that urocortin I postconditioning can regulate the expression of Bcl-2 and Bax and maintain cell viability through mitoK_ATP_ channel opening.

Several studies have indicated that drug postconditioning could significantly improve the function of the left ventricular [30]. Our cardiac function results showed that urocortin I could increase cardiac contractility, reduce left ventricular and systolic volume, and improve cardiac function during MIRI. We supposed that the protective effects of urocortin I postconditioning on isolated heart were related to the maintaining of normal respiratory function of mitochondria and the stability of cardiolipin content. In addition, urocortin I could inhibit the surge of ROS and the opening of mPTP and maintain the stabilization of MMP to inhibit the activation of mitochondrial apoptosis pathway. However, these effects of urocortin I were blocked by 5-HD, and the results also indicated that the protective effects on isolated heart were still related to the opening of mitoK_ATP_.

Some deficiencies and questions will be addressed in our future studies. Firstly, our experiment did not elucidate whether 5-HD alone had protective effects on isolated heart and cardiomyocytes, our subsequent experiments will detect the opening or closing state of mitoK_ATP_ during IRI/HRI. Secondly, ATP production and the apoptosis of cardiomyocytes were not measured in our experiment. In the follow-up experiment, we will further discuss the potential relationships between urocortin I postconditioning and these two indexes. Thirdly, our experiments did not test the metabolism of mitochondria, we will measure O_2_ consumption rate (OCR) and extracellular acidification rate (ECR) by Seahorse in our future study. In addition, hypothermia during IRI might has cardioprotective effect, and our study did not analyze the data at the end of ischemia.

In summary, we have identified that urocortin I postconditioning can sustain the normal transmission of electron transport chains and the normal function of oxidative phosphorylation through maintaining State3 and RCR and maintain the activity of respiratory enzymes such as Suc-OX, NADH-OX, and Cyt-OX. Besides, urocortin I can also inhibit the surge of ROS and maintain cardiolipin content to protect the integrity of the mitochondrial membrane. In addition, urocortin I can inhibit the opening of mitochondrial permeable transition pores and protect mitochondrial membrane potential to reduce the releasing of proapoptosis proteins. These effects of urocortin I postconditioning are related to the opening of mitochondrial ATP-sensitive potassium channel. Therefore, urocortin I may be a potential therapeutic drug to inhibit myocardial ischemia-reperfusion injury in patients with heart disease.

## Data Availability

The data used to support the findings of this study are available from the corresponding author upon request.

## References

[1] Korkmaz-Icöz S., Sun X., Li S. (2021). Conditioned medium from mesenchymal stem cells alleviates endothelial dysfunction of vascular grafts submitted to ischemia/reperfusion injury in 15-month-old rats. *Cell*.

[2] Jiang Y., Liu J., Peng W., Wang A., Guo L., Xu Z. (2021). Comparison of invasive blood pressure monitoring versus normal non-invasive blood pressure monitoring in ST-elevation myocardial infarction patients with percutaneous coronary intervention. *Injury*.

[3] Liu G., Zhang H., Hao F. (2018). Clusterin reduces cold ischemia-reperfusion injury in heart transplantation through regulation of NF-kB signaling and Bax/Bcl-xL expression. *Cellular Physiology and Biochemistry*.

[4] Vaughan J., Donaldson C. J., Bittencourt J. (1995). Urocortin, a mammalian neuropeptide related to fish urotensin I and to corticotropin-releasing factor. *Nature Cell Biology*.

[5] Lawrence K. M., Kabir A. M. N., Bellahcene M. (2005). Cardioprotection mediated by urocortin is dependent upon PKC*ε* activation. *The FASEB Journal*.

[6] Rademaker M. T., Charles C. J., Espiner E. A., Frampton C. M., Lainchbury J. G., Richards A. M. (2005). Four-day urocortin-I administration has sustained beneficial haemodynamic, hormonal, and renal effects in experimental heart failure. *European Heart journal*.

[7] Cong B. H., Wang L., Zhu X. Y., Li X., Liu B., Ni X. (2014). SGK1 is involved in cardioprotection of urocortin-1 against hypoxia/reoxygenation in cardiomyocytes. *Cardiology*.

[8] Díaz I., Calderón-Sánchez E., Toro R. d. (2017). miR-125a, miR-139 and miR-324 contribute to urocortin protection against myocardial ischemia-reperfusion injury. *Scientific Reports*.

[9] Lesnefsky E. J., Chen Q., Tandler B., Hoppel C. L. (2017). Mitochondrial dysfunction and myocardial ischemia-reperfusion: implications for novel therapies. *Annual Review of Pharmacology and Toxicology*.

[10] Wasmus C., Dudek J. (2020). Metabolic alterations caused by defective cardiolipin remodeling in inherited cardiomyopathies. *Life (Basel)*.

[11] Tam J., Hong A., Naranjo P. M. (2018). The role of decreased cardiolipin and impaired electron transport chain in brain damage due to cardiac arrest. *Neurochemistry International*.

[12] Chao H., Lin C., Zuo Q. (2019). Cardiolipin-dependent mitophagy guides outcome after traumatic brain injury. *The Journal of Neuroscience*.

[13] Halestrap A. P., Clarke S. J., Javadov S. A. (2004). Mitochondrial permeability transition pore opening during myocardial reperfusion—a target for cardioprotection. *Cardiovascular Research*.

[14] Jinsen L., Lele W., Wang X., Zhu J., Du J., Shen B. (2018). Detection of mitochondria membrane potential to study CLIC4 knockdown-induced HN4 cell apoptosis in vitro. *Journal of Visualized Experiments*.

[15] Liu C. N., Yang C., Liu X. Y., Li S. (2005). In vivo protective effects of urocortin on ischemia-reperfusion injury in rat heart via free radical mechanisms. *Canadian Journal of Physiology and Pharmacology*.

[16] Latchman D. S. (2001). Urocortin protects against ischemic injury via a MAPK-dependent pathway. *Trends in Cardiovascular Medicine*.

[17] Rasmo D. D., Signorile A., Santeramo A. (2015). Intramitochondrial adenylyl cyclase controls the turnover of nuclear-encoded subunits and activity of mammalian complex I of the respiratory chain. *Acta Biochimica et Biophysica Sinica*.

[18] Wilson D. F., Harrison D. K., Vinogradov A. (2014). Mitochondrial cytochrome c oxidase and control of energy metabolism: measurements in suspensions of isolated mitochondria. *Journal of Applied Physiology*.

[19] Li P. A., Hou X. L., Hao S. C. (2017). Mitochondrial biogenesis in neurodegeneration. *Journal of Neuroscience Research*.

[20] Kristiansen S. B., Henning O., Kharbanda R. K. (2005). Remote preconditioning reduces ischemic injury in the explanted heart by a KATPchannel-dependent mechanism. *Physiology*.

[21] Ghanian Z., Konduri G. G., Audi S. H., Camara A. K. S., Ranji M. (2018). Quantitative optical measurement of mitochondrial superoxide dynamics in pulmonary artery endothelial cells. *Journal of Innovative Optical Health Sciences*.

[22] Chu Q., Zhang Y., Zhong S. (2019). N-n-butyl haloperidol iodide ameliorates oxidative stress in mitochondria induced by hypoxia/reoxygenation through the mitochondrial c-Jun N-terminal kinase/sab/Src/reactive oxygen species pathway in H9c2 cells. *Oxidative Medicine and Cellular Longevity*.

[23] Masui K., Kashimoto S., Furuya A., Oguchi T. (2006). Isoflurane and sevoflurane during reperfusion prevent recovery from ischaemia in mitochondrial KATP channel blocker pretreated hearts. *European Journal of Anaesthesiology*.

[24] Grover G. J., Murray H. N., Baird A. J., Dzwonczyk S. (1995). The K_ATP_ blocker sodium 5-hydroxydecanoate does not abolish preconditioning in isolated rat hearts. *European Journal of Pharmacology*.

[25] Zhao Y., Guo Y., Chen Y., Liu S., Wu N., Jia D. (2020). Curculigoside attenuates myocardial ischemia-reperfusion injury by inhibiting the opening of the mitochondrial permeability transition pore. *International Journal of Molecular Medicine*.

[26] Patel P., Mendoza A., Robichaux D. J., Wang M. C., Wehrens X. H. T., Karch J. (2021). Inhibition of the anti-apoptotic Bcl-2 family by BH3 mimetics sensitize the mitochondrial permeability transition pore through Bax and Bak. *Developmental Biology*.

[27] Scarabelli T., Stephanou A., Rayment N. (2001). Apoptosis of endothelial cells precedes myocyte cell apoptosis in ischemia/reperfusion injury. *Circulation*.

[28] Boccalini G., Sassoli C., Formigli L., Bani D., Nistri S. (2015). Relaxin protects cardiac muscle cells from hypoxia/reoxygenation injury: involvement of the Notch-1 pathway. *FASEB Journal*.

[29] Buja L. M. (2022). Pathobiology of myocardial ischemia and reperfusion injury: models, modes, molecular mechanisms, modulation and clinical applications. *Cardiology in Review*.

[30] Chen W., Deng M., Wang H., Wang Y., Zhou W., Yu T. (2021). ROS-associated mechanism of different concentrations of pinacidil postconditioning in the rat cardiac Nrf2-ARE signaling pathway. *Molecular Medicine Reports*.

